# Closure of a large post-endoscopic full-thickness resection defect in the duodenum using a novel through-the-scope twin clip

**DOI:** 10.1055/a-2648-7419

**Published:** 2025-08-08

**Authors:** Yiying Qi, Aihong Yin, Hongyan Liu, Zhi Wei

**Affiliations:** 1 611300 Department of Gastroenterology, Shandong Second Provincial General Hospital, Jinan, China

A 46-year-old man was admitted to our hospital with a 2.5-cm submucosal tumor in the duodenal bulb ( Fig. 1 ). Enhanced computed tomography revealed a rounded abnormal enhancement in the duodenal bulb ( Fig. 2 ). Endoscopic ultrasound showed a heterogeneous hypoechoic lesion originating from the muscularis propria with both intraluminal and extraluminal growth ( Fig. 3 ). A submucosal injection was performed, followed by a mucosal incision around the intraluminal component of the tumor to expose the pseudocapsule.

**Fig. 1:**
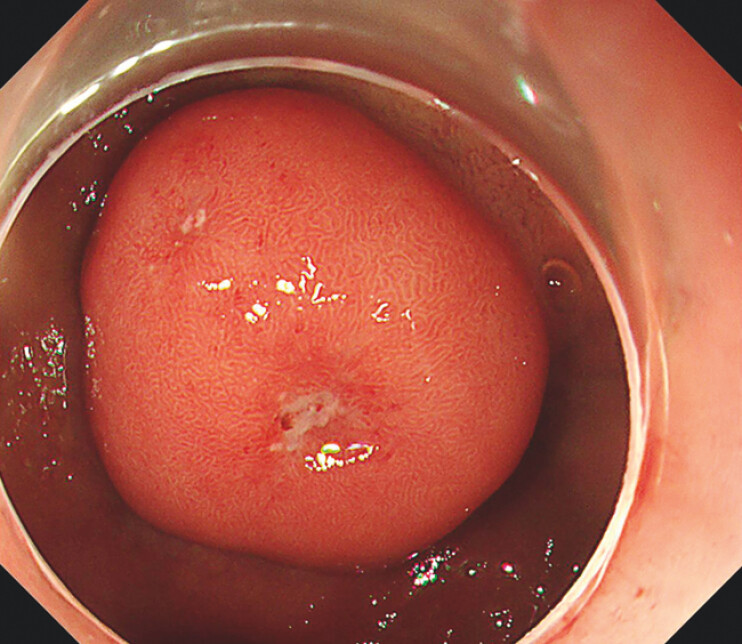
The tumor was located in the duodenal bulb.

**Fig. 2:**
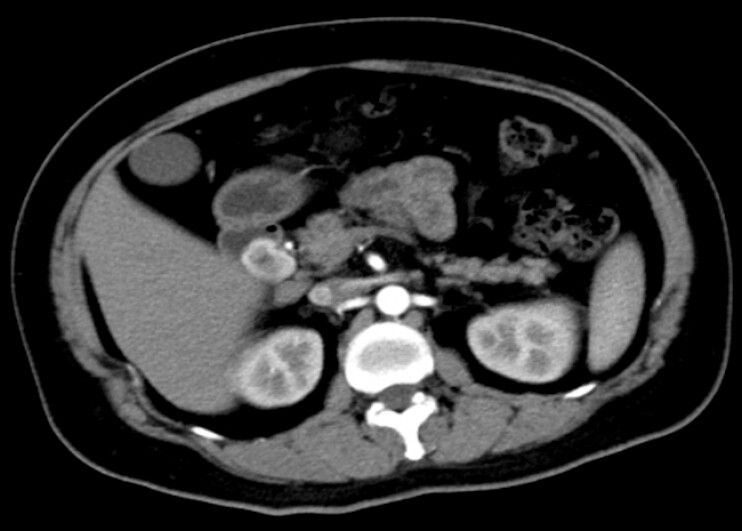
Enhanced computed tomography showed a round-like abnormal enhancement in the duodenal bulb.

**Fig. 3:**
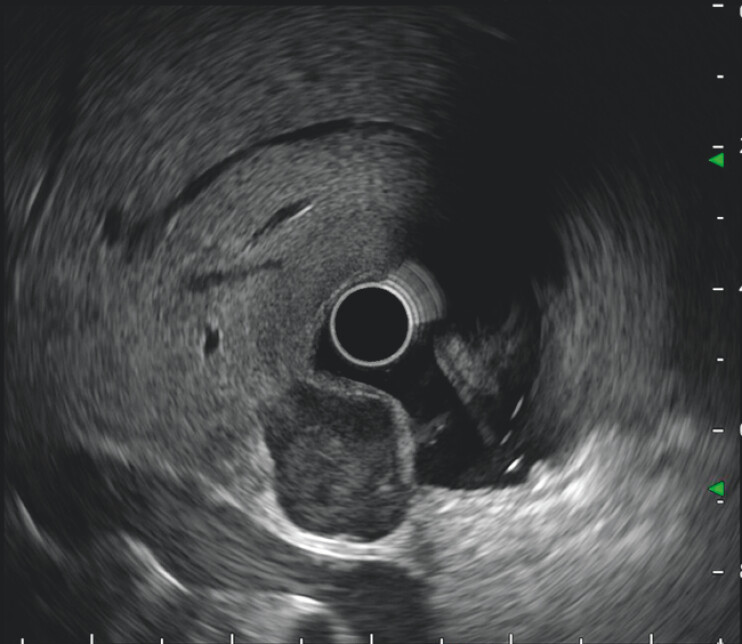
Endoscopic ultrasound showed a heterogeneous hypoechoic lesion originating from the muscularis propria.

Endoscopic full-thickness resection (EFTR) with 8-ring assisted traction was performed to remove the lesion, during which thick nourishing arteries were observed. The lesion was successfully resected ( Fig. 4 **a** ), with a wound size of approximately three cm ( Fig. 4 **b** ). A through-the-scope twin clip (TTS-TC) was used to close the full-thickness defect ( Fig. 5 **a** , Video 1 ). The TTS-TC was inserted through the biopsy channel of the endoscope, clamping one side of the wound while positioning the other side before releasing the TTS-TC. This process transformed the large defect into smaller ones. Subsequently, two TTS-TCs and several traditional through-the-scope clips were deployed to completely close the defect. Finally, surgical glue was sprayed on the surface of the wounds ( Fig. 5 **b** ). The patient fasted for three days and was discharged after eight days with no postoperative complications. Histopathological analysis revealed a low-risk gastrointestinal stromal tumor (GIST).

**Fig. 4:**
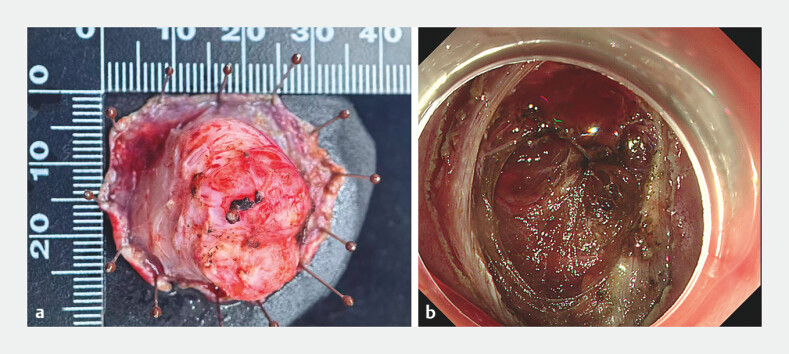
**a** The excised specimen. **b** The duodenal wall defect after the full-thickness resection.

**Fig. 5:**
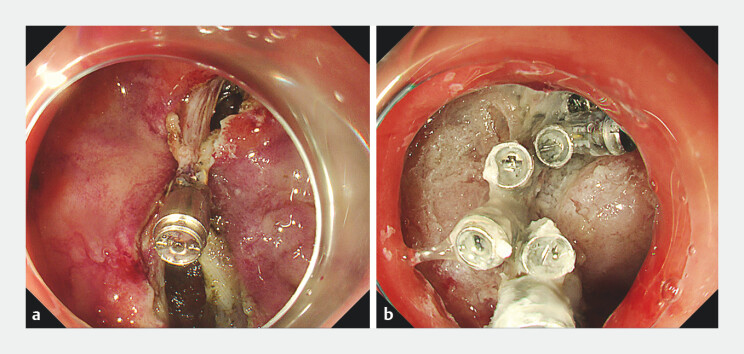
**a** The full-thickness defect sealed with a through-the-scope twin clip. **b** Complete sealing of wound.

A novel through-the-scope twin clip is used to close a large post-endoscopic full-thickness resection defect in the duodenum.Video 1

Tumors originating from the muscularis propria layer and adhering to the serosal layer can be resected through EFTR. Due to the unique anatomical location of the duodenum, closing large wounds remains a significant clinical challenge. Previous reports have documented the use of endoscopic suturing systems for closing wounds following EFTR of the duodenum 1 2 . We report the successful application of TTS-TC combined with surgical glue, which serves as an accurate, efficient, and safe method for sealing duodenal defects occurring during EFTR.

Endoscopy_UCTN_Code_TTT_1AO_2AO

## Notes

Endoscopy E-Videos https://eref.thieme.de/e-videos
*E-Videos* is an open access online section of the journal *Endoscopy* , reporting on interesting cases and new techniques in gastroenterological endoscopy. All papers include a high-quality video and are published with a Creative Commons CC-BY license. Endoscopy E-Videos qualify for HINARI discounts and waivers and eligibility is automatically checked during the submission process. We grant 100% waivers to articles whose corresponding authors are based in Group A countries and 50% waivers to those who are based in Group B countries as classified by Research4Life (see: https://www.research4life.org/access/eligibility/ ). This section has its own submission website at https://mc.manuscriptcentral.com/e-videos .
